# A Mixed-Rate Strategy on a Bilaterally-Synchronized Cochlear Implant Processor Offering the Opportunity to Provide Both Speech Understanding and Interaural Time Difference Cues

**DOI:** 10.3390/jcm13071917

**Published:** 2024-03-26

**Authors:** Stephen R. Dennison, Tanvi Thakkar, Alan Kan, Mario A. Svirsky, Mahan Azadpour, Ruth Y. Litovsky

**Affiliations:** 1 Waisman Center, University of Wisconsin-Madison, Madison, WI 53706, USA; srdennison@wisc.edu; 2 Department of Psychology, University of Wisconsin-La Crosse, La Crosse, WI 54601, USA; tthakkar@uwlax.edu; 3 School of Engineering, Macquarie University, Sydney, NSW 2109, Australia; alan.kan@mq.edu.au; 4 Department of Otolaryngology, New York University, New York, NY 10016, USA; mario.svirsky@nyulangone.org (M.A.S.); mahan.azadpour@nyulangone.org (M.A.)

**Keywords:** cochlear implants, research processor, binaural hearing, synchronization

## Abstract

**Background/Objective:** Bilaterally implanted cochlear implant (CI) users do not consistently have access to interaural time differences (ITDs). ITDs are crucial for restoring the ability to localize sounds and understand speech in noisy environments. Lack of access to ITDs is partly due to lack of communication between clinical processors across the ears and partly because processors must use relatively high rates of stimulation to encode envelope information. Speech understanding is best at higher stimulation rates, but sensitivity to ITDs in the timing of pulses is best at low stimulation rates. **Methods:** We implemented a practical “mixed rate” strategy that encodes ITD information using a low stimulation rate on some channels and speech information using high rates on the remaining channels. The strategy was tested using a bilaterally synchronized research processor, the CCi-MOBILE. Nine bilaterally implanted CI users were tested on speech understanding and were asked to judge the location of a sound based on ITDs encoded using this strategy. **Results:** Performance was similar in both tasks between the control strategy and the new strategy. **Conclusions:** We discuss the benefits and drawbacks of the sound coding strategy and provide guidelines for utilizing synchronized processors for developing strategies.

## 1. Introduction

Bilateral cochlear implants (BiCIs) are a successful treatment option for individuals with bilateral moderate-to-profound sensorineural hearing loss [1,2]. This type of treatment involves the implantation of an electrode array into each cochlea to stimulate the auditory nerve and provide a sense of sound perception. Bilateral processors lead to greater enjoyment of music [3] and substantial improvements in quality of life [4]. The main advantage of bilateral processors lies in their ability to significantly enhance sound localization accuracy and improve speech recognition in noisy environments. This improvement is facilitated by giving access to head shadow and binaural cues [5]. One of the biggest hurdles to delivering a full representation of binaural hearing (i.e., “hearing with two ears”) is the successful encoding of interaural time differences (ITDs) so that a listener with BiCIs can faithfully report the location of a sound source [6]. Two decades of work have revealed that BiCI listeners with adult-onset deafness show binaural sensitivity to constant amplitude low-rate stimulation (~100 Hz) presented at a single electrode [7]. Yet, patients with BiCIs have limited sensitivity to these cues in more complex acoustic environments, which explains why they experience difficulties locating sound sources compared to listeners with normal hearing (NH), particularly in noise [5,8]. 

NH listeners can utilize ITD cues, especially at low frequencies, to locate sounds in the horizontal plane [9]. The difficulties faced by BiCI users arise from two main issues: (1) unsynchronized bilateral clinical processors cannot guarantee that an ITD in the timing of pulses is well represented [7]; (2) clinical stimulation rates are typically greater than or equal to 900 pulses per second (pps) per channel [10,11,12], which can prevent clinical devices from accurately conveying ITDs in the timing of pulses [13]. BiCI listeners have previously shown the ability to use ITDs in the timing of pulses when the ITD cues are defined a priori and are explicitly encoded using research processors [14,15]. This sensitivity to ITDs in the timing of pulses has not been reliably demonstrated in real-time processing. Etiological factors such as age, cochlear implant experience, years of BiCI exposure, and the onset of deafness also play a complex role in sensitivity to ITDs, even with optimal encoding [16]. 

In a laboratory setting, BiCI listeners have demonstrated sensitivity to ITDs when deliberately encoded in the pulse timing of electrical stimulation using synchronized research processors [17]. These studies demonstrate the potential to overcome the constraints of unsynchronized clinical processors, allowing for the time-coordinated presentation of binaural cues to both ears. BiCI listeners are able to discriminate and lateralize (localize within the head) auditory sources using ITDs when stimuli are provided to single electrode pairs via synchronized, highly controlled desktop research processors [7,8]. However, BiCI listeners still vary drastically in their performance in ITD tasks and are far from achieving the same sensitivity as their NH counterparts [18]. While NH listeners show ITD discrimination thresholds as low as 10–20 μs for frequencies below 1000 Hz, BiCI listeners with adult-onset deafness have much higher ITD thresholds, ranging from 50–1000 μs when presented via synchronized research processors at low stimulation rates (~100–300 pulses per second, pps) [19]. Nevertheless, clinical stimulation rates are deliberately set much higher, generally exceeding 900 pulses per second (pps) per channel, aiming to better capture and convey speech envelope cues [10,11,12]. As a result, these rates are too fast to achieve optimal ITD sensitivity.

This issue of needing lower rates to encode timing differences has been partially addressed with the design of MED-EL’s fine structure processing (FSP) coding strategy and its successors, FS4 and FS4-p [20,21]. The FS4 strategy was designed to relay fine timing information from an acoustic waveform by introducing a pulse at each positive-going zero crossing in a channel’s band-pass filter output, leading to a repetition rate low enough to follow the instantaneous frequency. These low-rate pulses are delivered to no more than four apical-most channels of the electrode arrays. This processing method led to some improvements in subjective pitch perception [20]. However, the findings on improvements in ITD sensitivity are mixed. During binaural task assessments, the utilization of the FS4 strategy resulted in ITD thresholds ranging from 2.2 ms to 3.3 ms, in contrast to HDCIS, a high-rate-only strategy that produced no measurable ITD thresholds [22]. Notably, among the twelve listeners examined in [22], at least four demonstrated an enhancement from an initially non-measurable just-noticeable difference (JND) in the CIS strategy to an ITD JND threshold below 1.85 ms when employing the FS4 strategy. While measurable thresholds are a good outcome for any bilateral listener, ITDs of this magnitude are greater than the largest physiologically relevant ITDs for human sound localization (about 700–760 μs) [23]. This reveals that FS4 may not be able to provide ITD cues that are usable for real-world binaural tasks. 

To address the trade-off between ITD sensitivity and speech comprehension, researchers have explored explicitly encoding ITDs, either through the timing of individual pulses (pulse ITD) or the amplitude modulations of a pulse train (envelope ITD) [24]. Given that a single channel cannot simultaneously provide high and low rates, strategies like FS4 utilize “mixed rates” across channels. This approach employs some channels with high rates for representing rapid envelope changes, and others with low rates to enhance ITD sensitivity. Early attempts, similar to FSP and focusing on “peak derived timing” from low frequency channels, showed modest binaural improvements [8,19]. However, it is possible that the lack of explicitly encoded ITDs in pulse timing precluded any additional benefits. Recent studies leveraging controlled direct electrical stimulation have demonstrated the mixed-rate strategy’s effectiveness in binaural hearing tasks [14,15]. For instance, using even a single low-rate channel with explicitly-encoded ITDs amidst high-rate channels can significantly improve ITD sensitivity [25]. Together, these findings serve as proof of concept, affirming the feasibility of employing an across-channel mixed-rates strategy to achieve both ITD sensitivity and speech comprehension. 

Nevertheless, a persistent challenge is that the existing evidence supporting mixed-rates strategies relies on pre-processed stimulation patterns. Few practical implementations of these sound coding strategies have been attempted. Pre-processed stimulation patterns necessitate prior knowledge of the interaural time difference (ITD) for all stimuli. In real-world scenarios, ITDs are unknown, requiring estimation based on the available input. These calculations may require more processing time and power and are not guaranteed to be accurate. Consequently, previous “mixed rates” approaches only show the potential benefits of a mixed-rate strategy when the low and high rates have been tightly controlled and delivered. A real-time implementation is necessary to realize these potential benefits in realistic settings. We emphasize the need for real-time algorithms because the gold standard for evaluating new sound coding strategies should be the ability to run on a processor. 

Bilaterally synchronized processors are required for explicitly encoding precise ITD cues in the timing of stimulation pulses because unlinked processors cannot share timing information across the ears. Recently, the CCi-MOBILE, a bilaterally-synchronized research processor, has been shown to provide spatial cues to BiCI listeners [26]. The CCi-MOBILE enables simultaneous control of two implant devices. The CCi-MOBILE was used to measure BiCI listeners’ perception of lateralized sound sources with amplitude-modulated stimuli containing interaural level differences (ILDs) and envelope ITDs. Results showed that, in the presence of ILDs, envelope ITDs minimally affected lateralization responses in BiCI listeners. However, without the ILD, envelope ITD sensitivity ranged from 102–736 µs [26]. The focus of this work centers on the advancement of bilateral sound coding strategies that operate in tandem with synchronized processors. Our goal is to provide a working proof-of-concept of a novel mixed-rate sound coding strategy that can provide ITD cues through synchronized processors, without compromising speech information. This research draws inspiration from the late Dr. Loizou, specifically his groundbreaking work on the ciPDA, which resulted in the creation of the CCi-MOBILE that has enabled this work [27]. Dr. Loizou’s overarching aim for creating the ciPDA was to advance the development of bilateral sound coding strategies. 

The aim of this study was to perceptually evaluate a practical implementation of a mixed-rate sound coding strategy that can deliver explicitly encoded ITD cues via low-rate channels while maintaining speech intelligibility via high-rate channels. The effectiveness of this mixed-rate strategy was assessed through two psychophysical tasks: sound lateralization and binaural speech recognition in noise. This strategy was implemented using the CCi-MOBILE portable research platform [27] and was compared to the Advanced Combination Encoder (ACE) [12] and Continuous Interleaved Sampling (CIS) [28] sound coding strategies, neither of which explicitly encode ITDs. We hypothesized that, if participants were able to use explicitly encoded ITDs to lateralize a sound source, then a binaural benefit in speech-in-noise understanding would be observed. 

For lateralization, prior work has demonstrated that ITD cues explicitly encoded in the timing of pulses and delivered using a pre-processed direct stimulation method can yield good ITD sensitivity [15]. Therefore, it was expected that a mixed-rate strategy that can encode explicitly encoded low-rate pulse ITDs would lead to similar or improved ITD sensitivity as compared to an all-high-rate CIS reference strategy. It was hypothesized that the mixed-rate strategy would lead to effective lateralization due to the introduction of explicitly encoded ITDs in the timing of low-rate pulses. We further predicted that combining ITDs in the timing of low-rate pulses with ITDs in the envelopes of high-rate channels would lead to improved lateralization performance compared to only providing either of the ITD cues alone.

For speech recognition, the aim was to investigate the impact of the mixed-rate strategy on word recognition in a speech-in-noise task and whether there would be a binaural benefit when a low-rate pulse ITD was explicitly encoded via the sound coding strategy. A binaural benefit was measured using the binaural intelligibility level difference (BILD) [29] to understand whether a greater binaural benefit in speech understanding could be obtained using the mixed-rate strategy than with an all-high strategy. 

## 2. Materials and Methods

### 2.1. Participants

Nine bilateral-CI users were tested at the University of Wisconsin–Madison. All experimental procedures were approved by the Health Sciences Institutional Review Board of the university and followed best practices for testing with research interfaces as outlined in [30]. All participants were paid for their travel and time in the lab. The only inclusion criterion was the use of two Cochlear Corp. (Sydney, Australia) Nucleus 24 internal devices, with more than 1 year of experience with both implants. CNC word scores, which were used as a reference for past performance with everyday processors, were taken from a prior visit. If CNC word scores were not available, they were measured in the sound field according to best clinical practice prior to testing. See Table 1 for participant information.

### 2.2. Apparatus

All experiments were conducted with the CCi-MOBILE, a portable research platform developed at the University of Texas at Dallas [27]. The platform is a hardware platform that allows a personal computer or Android smartphone to process a microphone signal and stimulate Cochlear-branded cochlear implants. In this study, all processing was performed on a Microsoft Surface Pro tablet running the Windows 10 operating system (Redmond, WA, USA) and MATLAB version 2017a (Mathworks, Natick, MA, USA). The CCi-MOBILE, unlike clinically available processors, is bilaterally synchronized and capable of coordinating signal processing and stimulation across the ears [26]. While the CCi-MOBILE has microphones, they were not used in this experiment. Rather, digital stimuli with tightly controlled binaural cues were processed by the CCi-MOBILE in real-time. This setup allowed us to demonstrate the real-time processing capabilities of the proposed strategy while ensuring that known binaural cues were present in the input stimulus.

### 2.3. Processing Strategies

**Figure 1:**
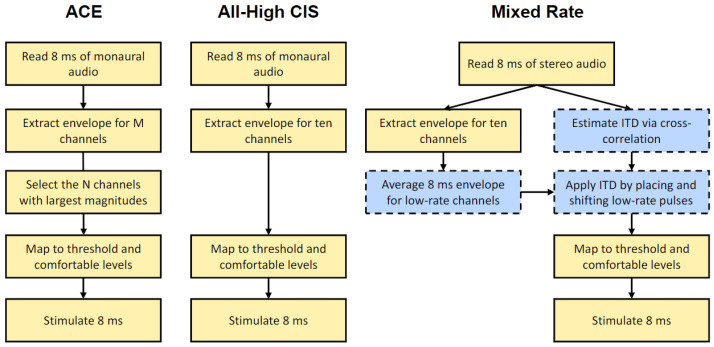
Schematic flowcharts for the three strategies discussed in this study. ACE = Advanced Combination Encoder, CIS = Continuous Interleaved Sampling.

The signal processing steps for the strategies tested in this study are visually shown in Figure 1. The experimental mixed-rate sound coding strategy tested in this study was inspired by the Continuous Interleaved Sampling (CIS) strategy [31,32]. Due to implementation limitations of the CCi-MOBILE and the overall stimulation rate limitations of Cochlear-branded devices, only 10 channels could be used in each ear to implement the mixed-rate strategy. Our implementation provides an overall stimulation rate of 10,000 pps across all channels. The full algorithm for the mixed-rate strategy is as follows:

1. Buffering. Input audio is stereo and at a sampling frequency of 16,000 Hz. On the CCi-MOBILE, incoming audio is buffered as 8 ms frames. The buffered frames are processed as overlapping blocks of 128 samples, with a hop size of 1 ms and 7 ms of overlap in each block.
2. Windowing. A 128-point Hann window is applied to each block. The Hann window is calculated as:
  w(n) = 0.5 − 0.5 cos(πn/2), for n = 0 to 127(1)
3. Fast Fourier Transform (FFT). A 128-point FFT is then applied to each block. Only the first 65 bins are retained, discarding bins for negative frequencies.
4. Magnitude estimation. The complex values in the transformed frame are multiplied by their complex conjugates to estimate the power in each frequency bin. A frequency-weighted scaling is applied based on how many channels are active. The frequency bins are consolidated into ten frequency channels (see Table 2 for frequency allocations). The square root of each entry in this matrix is then calculated to provide an estimate of the channel energy.
5. ITD estimation. The delay that maximized the cross-correlation of left and right buffered frames from Step 1 above is used as an estimate of the ITD in the input signal at that particular point in time. The delay is rounded up to the nearest multiple of 100 µs, the reciprocal of the overall stimulation rate of 10,000 pps across all channels.
6. ITD encoding. ITDs are encoded once per 8 ms frame on each channel designated for low-rate stimulation. Depending on the delay estimated in the ITD estimation step, either the left or right pulses are delayed to encode the ITD. Because Cochlear devices can only stimulate one electrode at a time in each ear, any high-rate pulses that overlap in time with the low-rate pulses are removed. If there is an ITD of 0 μs, pulses of the two implants will be simultaneously scheduled in the low-rate channels. The amplitude of the low-rate pulses is the average energy over the entire 8 ms frame for that channel.
7. Dynamic range compression. The amplitude values are then normalized between an arbitrary base level and saturation level. The normalized values are then compressed using a logarithmic function and transformed to the proper current levels for each channel based on the Threshold (T) and Comfortable (C) levels in each patient’s clinical MAPs.

Any of the ten channels could be designed as high- or low-rate channels in this implementation. As such, the mixed-rate strategy in this experiment had five channels set to 125 pps and five channels set to 1000 pps, interleaved along the electrode array (see Table 2 for low- vs. high-rate channels). The interleaved pattern was selected for this study so that ITD information would be provided at different locations all along the electrode array. A similar configuration yielded lower median JNDs than strategies with only high rates in previous studies [15,25]. ITDs were not explicitly encoded in the timing of pulses in the high-rate channels. We judged that it was highly unlikely that these arbitrary ITDs would compromise the ITDs provided on low-rate channels because sensitivity to ITDs would be poor at 1000 pps due to the rate limitations the BiCI users experience (e.g., [7,18]). Participants were tested using the T and C levels that were programmed by their clinician. T and C levels were not re-measured for 125 pps stimulation rates. An implementation of the Advanced Combination Encoder (ACE) [12,27] was also tested to verify speech understanding in quiet.

### 2.4. Stimulus

For lateralization, stimuli were generated using custom-written software running on version 2018a of MATLAB (Mathworks, Natick, MA, USA) at a sampling rate of 16 kHz, by summing ten 300 ms sinusoids corresponding to the center frequencies of the analysis channels of interest. The indices for the electrodes targeted were 2, 4, 6, 8, 10, 12, 14, 16, 20, and 22. These electrode indices were chosen because their center frequencies, which are determined by taking the average frequency of one or more FFT bins, most closely matched the center frequencies of the analysis channels shown in Table 2. If the mixed-rate strategy was used, low-rate electrode indices were 4, 8, 12, 16, and 22 (see Table 2). If one of these pairs of electrodes was deactivated in the participants’ clinical MAP in either ear, then the nearest electrode pair was used for that individual. The same channel numbers were used in both ears. Stimuli were calibrated by scaling the digital signal so that the peaks of the electrical stimulation were within 70–80% of the electrical dynamic range for each participant at 1000 pps. Electrical stimuli were streamed to participants by processing the acoustic stimulus through a sound coding strategy in MATLAB and then writing to the internal implant device using the CCi-MOBILE.

**Figure 2:**
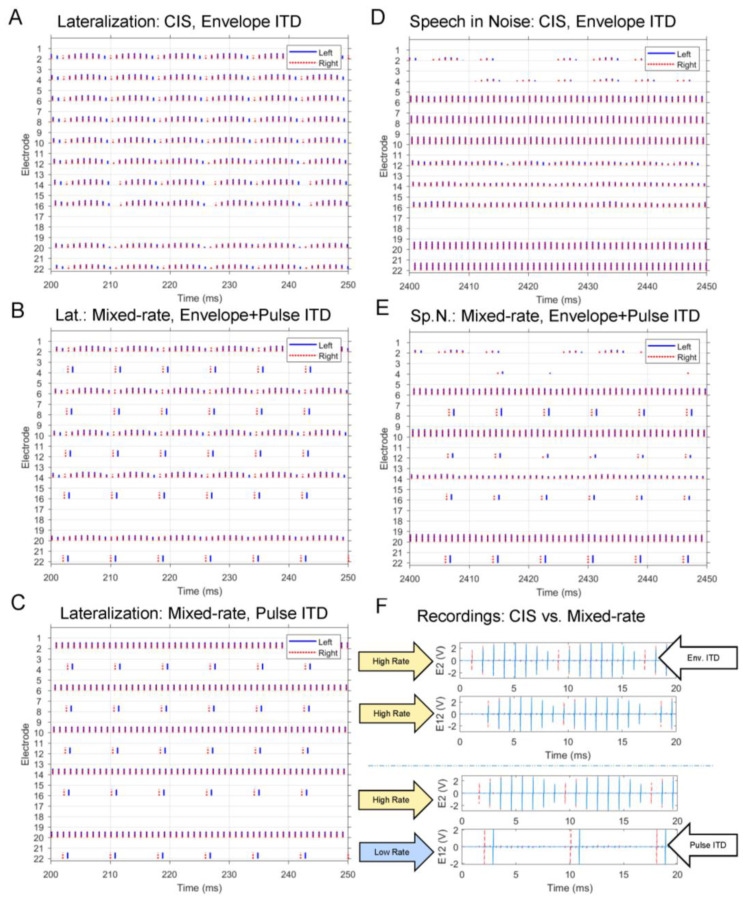
Electrodograms and processor output recordings for the stimuli used in the study. (**A**–**C**) depict lateralization (Lat.) stimuli with a +800 μs ITD. (**D**,**E**) depict speech-in-noise (Sp.N.) stimuli, the CNC word “bean”, with a +800 μs ITD at +3 dB SNR. Electrodograms show only 50 ms of the total stimulation pattern to allow for visualization of ITDs. (**F**) shows example recordings of processor outputs for comparing between (**A**,**B**). All maps used in these examples had ten channels with T and C levels of 100 and 200, respectively. The electrical stimulation output from the CCi-MOBILE was recorded at a sampling rate of 250 kHz per channel using a National Instruments USB-6343 data acquisition card (NIDAQ) connected to an Implant-in-a-box containing a CI24RE electrode array. Due to the limited sampling rate of the NIDAQ, left–right pairs of electrodes were measured two at a time, and the full electrodogram was reconstructed via the time-alignment of a common pair of electrodes in all measurements.

Three different test conditions were created for lateralization: (1) sinusoids with a 125 Hz amplitude modulation, to be processed using the all-high CIS strategy, (2) an identical signal to (1) but processed using the mixed-rate strategy, and (3) sinusoids with no amplitude modulation, to be processed using the mixed-rate strategy (see Table 3 for a summary). Conditions (1) and (2) were planned to directly compare performance between the CIS and mixed-rate strategies, while condition (3) was intended to account for the contribution of low-rate pulses without any amplitude modulation on the high-rate channels. For all three test conditions, ITDs were applied to these acoustic stimuli by delaying the overall waveform in the left or right channel by 800 μs. Low-rate pulses were timed to coincide with the onsets of the 125 Hz envelope modulation on the high-rate channels. These test conditions provided the following cues to participants: (1) envelope ITDs in all ten channels provided by the all-high CIS strategy, (2) envelope ITDs in the five high-rate channels and pulse ITDs in the five low-rate channels provided by the mixed-rate strategy, and (3) pulse ITDs in the five low-rate channels provided by the mixed-rate strategy, with no envelope modulations on the high channels. Thus, in this portion of the study, there was one condition using the all-high CIS strategy and two conditions using the mixed-rate strategy. Examples of each lateralization stimulus are depicted in Figure 2.

For speech testing, stimuli were recordings stored in WAVE files, processed in real time, and streamed to listeners via the CCi-MOBILE. Stimuli were a closed set of 30 one-syllable Consonant–Nucleus–Consonant (CNC) words spoken by a male talker. When testing speech recognition in noise, noise tokens were speech-shaped noise (SSN) matched to the CNC corpus spoken by the same male talker. The root–mean–square energy over the entire target stimulus duration was equalized across all stimuli. For the CNC words, the target was preceded by 1.55 s by the word “Ready”, spoken by the male talker. In noise conditions, the noise preceded and succeeded both “Ready” and the target word by 400 ms. The target speech was presented bilaterally at a comfortable loudness in one of four conditions: Quiet, −3, 0, or + 3 dB SNR. Three SNR conditions were tested because it was unclear, a priori, which SNR condition might reveal a binaural benefit. In every noise condition, a 0 μs interaural time difference (ITD) was applied to the speech-shaped noise (SSN) masker. The binaural cue conditions included “Zero ITD”, where both the masker and target word were presented with a 0 μs ITD, and “Non-Zero ITD”, featuring the masker presented at 0 μs ITD and the target word at +800 μs ITD. As such, seven stimulus categories were created: Quiet, −3 dB SNR Zero ITD, −3 dB SNR Non-Zero ITD, 0 dB SNR Zero ITD, 0 dB SNR Non-Zero ITD, +3 dB SNR Zero ITD, and +3 dB SNR Non-Zero ITD. All stimuli were tested with three sound coding strategies implemented with the CCi-MOBILE research platform: ACE, CIS, and mixed-rate; see Table 4. It should be noted that CI sound processing was performed on the sound mixtures, and not just clean speech. Examples of speech-in-noise stimuli are also depicted in Figure 2.

### 2.5. Experimental Procedure

Loudness balancing and the centering of stimuli, prior to the application of the ITD, were matched before the start of each experiment. Participants were instructed to first match the perceived loudness of each condition by adjusting the overall volume. Then, participants used a separate left–right visual slider to change the right–left balance until they perceived each sound as located in the center of their head. Adjustments were controlled by participants via a custom MATLAB graphical user interface (GUI) that controlled stimulus presentation and changed the overall loudness. For the lateralization task, participants were shown a GUI with a loudness slider and a centering slider for each of the three conditions that were tested; stimulus presentation was controlled with a “Play” button for each sound. For the speech task, participants were shown a GUI with a loudness slider and a centering slider for each of the three strategies tested; the stimulus was the word “Goose”, and presentation was controlled with a separate “Play” button for each strategy. 

Participants always completed lateralization before speech testing. During the lateralization experiment, participants used a computer screen and a mouse to indicate the perceived location of a sound in their head by clicking on an image of a face [33,34]. Responses were recorded by the software as integers between −50 and +50, with negative and positive responses representing left and right locations, respectively. After centering and loudness balancing, but before testing, participants were trained with the software by listening to and reporting the locations of perceived lateralized images with a ±5 dB interaural level difference (ILD). For lateralization testing, participants listened to and reported the perceived lateralized image of a single interval of the three stimulus conditions described in Table 3 with ITDs of either −800 or +800 μs. Each condition and ITD combination was tested 20 times in total, leading to 120 trials per participant. Testing was divided into four blocks, where each block contained five repetitions of all stimulus conditions and ITDs, presented in a fully random order. Breaks were provided when the participant requested, and testing took approximately an hour.

For speech testing, as with lateralization, the loudness and centeredness of an example word with 0 μs ITD processed by ACE, CIS, and mixed-rate strategies was subjectively matched before the start of the speech experiment. Participants were instructed to first match the perceived loudness of each condition by adjusting the overall volume via a GUI that controlled stimulus presentation and changed the overall loudness. Then, participants used a left–right slider to change the right–left balance until they perceived each sound as located in the center of their head. Participants used a similar GUI as with the lateralization stimulus to balance an example speech stimulus word.

Participants listened to a list of 30 CNC words presented in random order. Each block presented the same closed-set list of thirty words; only the strategy, noise condition, and binaural cue condition were varied, as well as a new randomization of the word order. Each listener was presented with a different word list in a random order. A total of 15 blocks were completed. The first three blocks were in quiet, with ACE first, then CIS, then the mixed-rate strategy. This was then followed by the remaining 12 speech-in-noise condition blocks which were presented in a randomized order, counterbalanced across participants. There were twelve speech-in-noise conditions, with CIS and mixed-rate strategies tested for three SNRs and two binaural configurations. Testing took approximately two hours including breaks.

### 2.6. Analysis

The lateralization range was calculated as the difference in means for left and right ITDs for each condition. ITD sensitivity (d’) was calculated by dividing the lateralization range by the pooled standard deviation for left and right lateralization responses. ITD d’ for each condition was compared using a mixed-effects model in R (version 4.1.0) using the “lme4” package (version 1.1-27.1) with the “lmer” function (model: Response~1+Condition+(1|ID)). Planned post hoc tests were used to compare the three conditions if the main model revealed an effect due to condition. Assumptions of normality and equal variances were checked using the “car” package (version 3.1-0) with the “shapiro.test” and “leveneTest” functions, respectively.

For speech testing, the percentage of words correctly identified was calculated for each participant, strategy, and SNR. First, to understand the impact of the mixed-rate strategy on speech understanding in quiet, a nonparametric Friedman test was used to evaluate whether any significant differences were observed due to strategy tested. Second, to understand the impact of introducing ITD information on speech understanding, binaural intelligibility differences (BILDs) were calculated for the CIS strategy and the mixed-rate strategy at each SNR for each participant:

BILD = (% correct with nonzero ITD) − (% correct with zero ITD) (2)

BILDs for each strategy were compared using a mixed-effects model (BILD~1+Strategy*SNR+(1|ID)) in R using the same packages as the lateralization data.

## 3. Results

### 3.1. Lateralization

**Figure 3:**
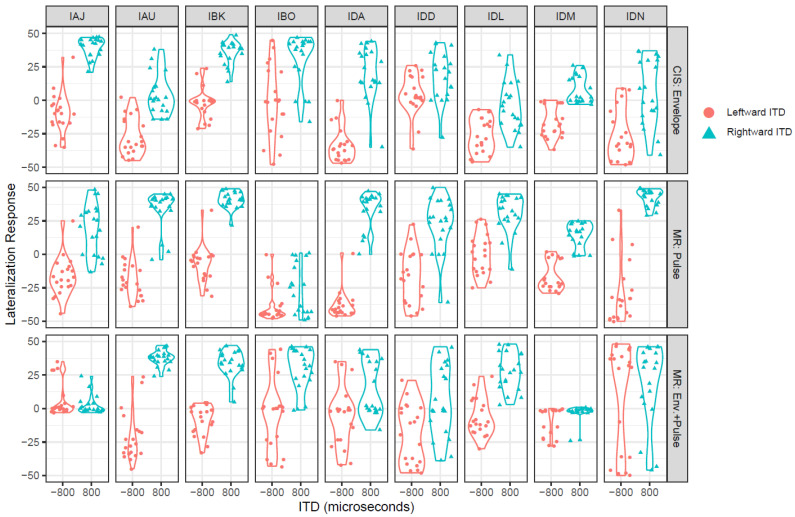
Lateralization responses for each listener. Each data point represents the response to one trial. Responses with negative or positive values indicate that the listener perceived the sound as lateralized to the left or right side of the head, respectively.

**Figure 4:**
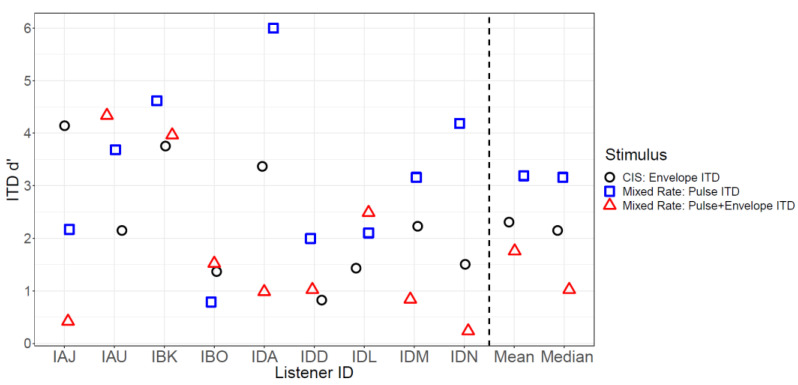
Summary of lateralization data showing interaural time difference (ITD) d’ scores for each processor condition.

Figure 3 shows the raw lateralization data for each participant in each stimulus condition: envelopes only (strategy: CIS), pulse timing of low-rate pulses only (strategy: mixed-rate), or both envelopes and low-rate pulse timing (strategy: mixed-rate). Lateralization range and ITD d’ were highly correlated (ρ = 0.90), so lateralization range was not analyzed. Figure 4 shows the average ITD d’ scores for each condition. Across all conditions, the average ITD d’ was 2.3. Mixed-effects ANOVA revealed no significant differences in ITD d’ due to condition (*F* [2, 16] = 2.9, *p* = 0.08). The intercept, representing the mean ITD d’ across all conditions and listeners, was found to be significantly different from zero (t [21.6] = 5, *p* < 0.001) using Sattherthwaite’s method. Mean ITD d’ was 1.7 for the envelope and pulse ITD provided by the mixed-rate strategy, 2.3 for the envelope ITD provided by the CIS strategy, and 3.1 for the pulse ITD provided by the mixed-rate strategy.

### 3.2. Speech Testing

**Figure 5:**
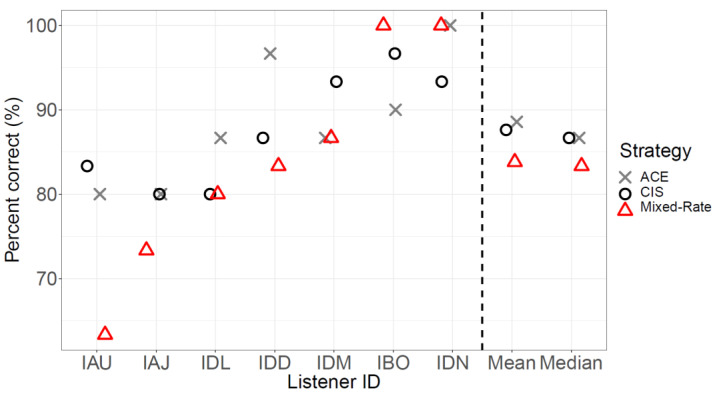
Word recognition scores for speech in quiet for each participant.

Figure 5 shows word recognition scores in quiet for each participant and their performance when listening with each of the three strategies. Median speech scores were 86.7%, 90.0%, and 83.3% for ACE, CIS, and mixed-rate strategies, respectively. A non-parametric Friedman test revealed significant differences across strategies (χ^2^ = 6.95, *p* = 0.03). Post hoc Wilcoxon signed-rank tests revealed no significant difference in performance between the ACE and CIS strategies (*p* = 0.76) and no significant difference in performance between ACE and mixed-rate strategies (*p* = 0.067). However, there was a significant difference between the CIS and mixed-rate strategies (*p* = 0.036).

**Figure 6:**
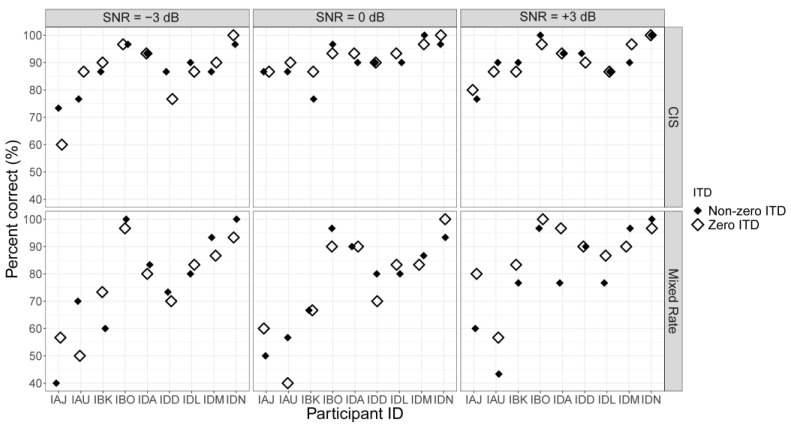
Word recognition scores for speech in noise for each participant. Triangles indicate results with zero ITD and circles indicate results with non-zero (+800 μs) ITD.

**Figure 7:**
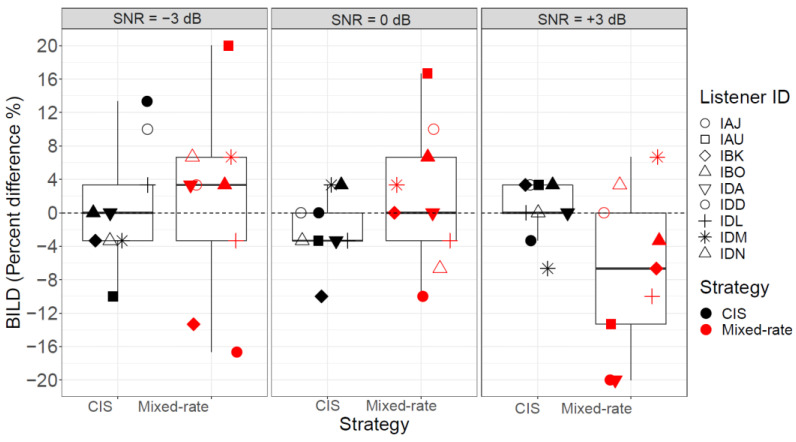
BILD showing the difference in performance between the “Non-zero ITD” and “Zero ITD” conditions. Data is shown for all SNRs (−3, 0 and +3 dB) and both the CIS and mixed-rate strategies. Values greater than 0-BILD indicate an improvement in the non-zero-ITD condition.

Figure 6 displays the word recognition scores for speech in noise across all participants. Raw word recognition scores for speech-in-noise scores were not analyzed and were transformed as in Equation (2) to calculate BILDs. Figure 7 shows the BILD (difference in performance between the non-zero-ITD and zero-ITD conditions). The BILD data passed the normality assumption (*p* = 0.08) and the equal variances assumption (*p* = 0.16). Mixed-effects ANOVA revealed no significant differences in BILD due to Strategy (*F* [1, 45] = 0.33, *p* = 0.57) or SNR (*F* [2, 45] = 1.78, *p* = 0.18), and no significant interaction between Strategy and SNR (*F* [2, 45] = 2.89, *p* = 0.07). There was a trend towards significance in the interaction term, likely reflecting the higher BILD for the mixed-rate strategy than the CIS strategy at −3 dB SNR. Thus, future studies should probably focus on this SNR level to further understand the binaural benefit of the mixed-rate strategy.

### 3.3. Relationship between Lateralization and Speech Data

The potential relationship between lateralization and the binaural benefit of speech understanding in noise was examined by calculating Spearman’s rank correlation. We chose to analyze only the −3 dB condition because it was the condition with the largest median BILD. Spearman’s rank correlation was computed to assess the relationship between ITD d’ and BILD performance in the CIS strategy. The correlation between the two variables was positive but not statistically significant (*r*(7) = 0.44, *p* = 0.23). A second Spearman’s rank correlation was computed to assess the relationship between ITD d’ for envelope and pulse ITD and BILD performance in the mixed-rate strategy. Again, the correlation was positive but not statistically significant (*r*(7) = 0.25, *p* = 0.51).

## 4. Discussion

The aim of this study was to evaluate a mixed-rate CI sound coding strategy that could operate in real-time on a bilaterally synchronized mobile CI research platform. We investigated whether this strategy could provide both explicitly encoded ITD cues via low-rate channels and speech perception via high-rate channels. Lateralization was measured using three stimulus conditions in order to compare the mixed-rate strategy to a CIS reference strategy using all-high rates, and to understand the contributions of the low-rate pulse ITDs and envelope ITDs to the lateralization of sounds. Speech understanding in quiet and in noise was evaluated to test the viability and benefits of the mixed-rate strategy for conveying both word recognition and ITDs, as compared to an all-high-rate CIS strategy.

In the lateralization experiment, participants demonstrated the ability to lateralize ITDs in all three stimulus conditions. However, not all listeners lateralized a large ITD to the side of the head; rather, they perceived large ITDs as being closer to the center of their heads, which was consistent with [35]. ITD sensitivity was calculated as an ITD d’ score for each condition and was not significantly different across stimulus conditions. This was consistent with research suggesting that both envelope ITDs and pulse ITDs can yield similar ITD thresholds for envelope modulation rates near 100 Hz [36]. Our hypothesis led to the prediction that there would be greater ITD sensitivity with the mixed-rate strategy used to encode a stimulus with envelope modulations than the CIS strategy. This might occur because the mixed-rate strategy could effectively provide both low-rate pulse interaural time differences (ITDs) and envelope ITDs, whereas the CIS strategy lacked the explicit encoding of low-rate ITDs. Having both types of ITDs encoded provides greater redundancy, especially when one of these cues is potentially masked, such as in a noisy situation. Performance was greater with both mixed-rate strategies that provided ITDs in the timing of pulses than with the CIS strategy which can only provide ITDs in the envelope of pulse trains. Performance was best with the testing condition that only delivered pulse ITDs and where modulations were not applied to the stimulus, but this difference was not significant. For some participants, the combination of envelope and pulse ITD seemed to be detrimental to lateralization. Performance was not significantly statistically different whether ITDs were delivered in low-rate pulse ITDs or envelope modulations on high-rate pulse trains. This is consistent with evidence from an animal model that showed that neurons in the inferior colliculus are well tuned to respond to ITDs both in the envelope and the fine structure of electrical stimulation [37,38]. One limitation of our approach was only testing one magnitude of ITD, a choice made to keep the number of trials down due to time restrictions on human testing during the COVID-19 pandemic. However, future studies should consider testing a range of ITDs to observe the benefits of the mixed-rate strategy at much smaller ITDs to evaluate the impact of pulse ITDs in such a manner. In addition, testing in noise should be considered, to observe whether the electrically stimulated auditory system can use both types of ITD cues to improve lateralization judgement when signal-to-noise conditions are poor.

It is encouraging that the condition with the highest average performance was a mixed-rate strategy condition where pulse ITDs were available. However, the perceptual findings indicated that not all participants performed best in the lateralization task using the mixed-rate strategy, and the worst condition overall was the condition combining envelope ITDs and pulse ITDs. One reason for lower performance in this condition could have been that the electrode pairs that were stimulated across the ears had interaural place-of-stimulation mismatch, as reduced frequency matching across the ears reduces ITD sensitivity [39].

For speech testing, performance in quiet was comparable across all strategies for all participants. Although there was a significant difference in performance due to strategy tested, the difference in means was only 6%, and listeners reported a noticeable difference in the clarity of the words when listening to the different strategies, especially with the mixed-rate strategy. Participants described the quality of the different sound coding strategies in several ways. One participant (IBO) described speech processed using the mixed-rate strategy as higher-pitched, while another participant (IAU) described speech processed using the mixed-rate strategy as lower-pitched when compared to ACE. Participants generally found ACE to be the most similar to their everyday listening, with the most clarity. The mixed-rate strategy was qualitatively described using words such as “muffled”, “growling”, “nonchalant”, and “garbled”, to capture the range of individual experiences.

The result that speech scores were similar across strategies is consistent with the findings of Churchill et al. [14]. In this study, only one participant, IAU, performed more poorly with the use of mixed rates as compared to CIS, dropping by 20%. However, this participant might have encountered challenges in adapting to the new strategy within a brief acclimatization period. Future studies should allow participants several hours to acclimatize to new strategies before testing. Despite this, the current findings offer promise for the ongoing adoption of mixed rates as an alternative speech sound coding strategy compared to a CIS or ACE strategy, because using mixed rates does not seem to significantly compromise performance in quiet environments. When noise was added, word recognition performance with mixed rates yielded comparable performance between the two ITD conditions for most listeners. That is, even though the speech envelope was degraded by noise in half the channels and there was a time difference across the ears, word recognition was still good at the SNRs tested.

To evaluate the impact of the ITD in the speech-in-noise task, BILD was assessed by comparing the difference in performance in a condition with a zero ITD vs. a non-zero ITD across the CIS and mixed-rate strategies. On one hand, it is encouraging that, on a group level, BILDs were not significantly less than zero, indicating that the introduction of an ITD via changes in low-rate pulse timing did not disrupt speech understanding. On the other hand, the lack of a group level positive BILD also indicates that the mixed-rate strategy was not effective at providing binaural benefits to all participants. However, on an individual level, it is notable that several participants showed large BILDs. This finding suggests that BILDs are indeed possible to achieve with the mixed-rate strategy, but that further work is needed to understand why only some participants experienced a benefit and how it might be possible to achieve such benefits at various SNRs. Future studies could aim to test a wider range of SNRs that would reveal greater improvements due to the ITD from the mixed-rate strategy.

Further, the lack of binaural benefits may be explained here due to other factors, such as the masker type and/or task type. For instance, Ref. [24] showed that ITDs delivered to a speech stimulus using a novel signal-processing algorithm, specifically designed for bilateral-CI users to enhance sound localization in noise, can produce perceived lateralization. This strategy duplicates a monophonic electrode pulse pattern and applies natural or artificial ITDs or ILDs based on the estimated direction of the dominant sound source, using their “PP” strategy, which was a combined version of the fundamental asynchronous stimulus timing (FAST) strategy [40], peak-derived timing strategy (PDT, see [8]), and the fine structure processing (FSP) coding strategy [20]. Further, they found that, while the introduction of the PP strategy improved performance in the lateralization task, it decreased overall speech perception compared to a CIS strategy. Thus, it is important to acknowledge that the increases in binaural benefits in a sound coding strategy have the potential to easily impact speech perception negatively. Ref. [22] found that ITD sensitivity in seven MED-EL users did not correlate to BILDs, which is similar to the results presented here. Our results, when considered alongside the findings from the study by [24], imply that enhancements in lateralization achieved through low-rate pulsatile pulse interaural time differences (ITDs) may not universally translate into binaural advantages for speech comprehension in noisy conditions. Addressing the intricate challenge of speech unmasking in noise is likely to necessitate the integration of multiple approaches and the exploration of the optimal customization of the mixed-rate strategy for individual users.

It is still unclear how best to achieve the optimal balance between the number of high-rate channels for effective speech information and low-rate channels for sufficient ITD sensitivity. Our current approach demonstrates that a minimal number of speech channels is sufficient for closed-set speech understanding. However, most CI recipients likely have more than ten channels available. For Cochlear electrode arrays, as many as 22 electrodes may be active, meaning that there are 231 possible permutations of low- and high-rate channels. An exhaustive investigation of every possible option is not practical for individual listeners, but future studies could guide the delivery of ITD cues by selecting the most sensitive regions of the cochlea for each listener.

There are several limitations to consider when interpreting and generalizing the results of these studies. First, ILDs were considered to be set to 0 dB, as is common in many investigations of the ITD sensitivity of CI participants [14,18]. This was achieved by having participants subjectively center example stimuli [33]. The presence of even a “zero magnitude” ILD likely influenced lateralization, as ILDs appear to be the dominant cue for localization and lateralization for CI listeners [6,41,42]. It will be important to consider the interactions of ILDs and ITDs, as the mixed-rate strategy, for example, does not currently explicitly encode ILDs on its low-rate channels but may introduce ILDs in the envelopes of all channels. Future implementations could include explicit encoding of ILDs. Second, the overall stimulation level of the stimuli may have impacted ITD sensitivity in both tasks for some listeners. T and C levels were not remapped for the 125 pps channels because of time constraints. However, using the T and C levels intended for clinical stimulation rates of 900 pps while using stimulation rates of 100 pps may have impacted the audibility of the ITD cues for some listeners. If the 1000 pps C levels were too low on low-rate channels, the overall current level may not have been sufficient for good ITD sensitivity [17]. Third, we did not take into consideration the impact of ILDs on performance. BiCI users are familiar with ILDs from their everyday processors and may expect ILDs to produce lateralized sounds. One recent study explored the enhancement of ILDs using an artificial current-versus-angle function to modify the levels delivered by the contralateral CI’s basal electrodes. This resulted in improvements in sound source discrimination on the frontal horizontal plane for some BiCI users [43].

Finally, a clear limitation of this study was its small and heterogeneous sample size. Our goal was to explore the practical application of a mixed-rate sound coding strategy, assessing the theory of integrating high and low stimulation rates across two tasks. As such, this has allowed us to generalize the viability of direct electrical stimulation to the real-time processing of ITDs. However, it is important to be cautious in extending these findings to all BiCI users and mixed-rate implementations. To overcome this limitation, future research should aim to include a larger, more representative sample of BiCI users.

Despite these limitations, the results presented here demonstrate the opportunity to develop and test binaural sound coding strategies for BiCI users. The real-time mixed-rate strategy, as tested here, can provide low-rate ITDs to participants, and produces perceptual results that are consistent with prior literature evaluating the mixed-rate strategy under more-controlled stimulus conditions. Future research is needed to optimize or individualize mixed-rate strategies and validate the benefits of providing low-rate ITDs to BiCI participants in real-time.

## Figures and Tables

**Table 1:** Participant information. CNC = Consonant–Nucleus–Consonant. pps = pulses per second. Participant IDs are anonymized labels. Preferred ear was determined by asking each participant which ear they favored in everyday listening.

Participant ID	Age	Years Bilaterally Implanted	Preferred Ear	Bilateral CNC Word Score in Sound Field	Best ITD JND at 100 pps (µs)
IAJ	76	18	Right	70%	240
IAU	72	15	Left	53%	269
IBK	82	13	Left	90%	58
IBO	57	14	Right	84%	100
IDA	55	8	Left	84%	438
IDD	23	8	Right	78%	610
IDL	67	4	Right	74%	303
IDM	44	9	Left	74%	287
IDN	20	9	Right	80%	N/A

**Table 2:** Frequency allocation information for input and output channels of the sound coding strategies. The frequency mapping to electrodes was chosen to be close to the clinical mapping frequency allocation table in order to maintain reasonable word recognition abilities using the novel maps with minimal adaptation. CIS = Continuous Interleaved Sampling, pps = pulses per second.

Analysis Channel	Electrode Array Index	Analysis Channel Low-Freq. (Hz)	Analysis Channel High-Freq. (Hz)	Rate of Stimulation, CIS (pps)	Rate of Stimulation, Mixed-Rate (pps)
1	22	188	438	1000	125
2	20	438	688	1000	1000
3	16	688	1063	1000	125
4	14	1063	1438	1000	1000
5	12	1438	1938	1000	125
6	10	1938	2563	1000	1000
7	8	2563	3438	1000	125
8	6	3438	4563	1000	1000
9	4	4563	6063	1000	125
10	2	6063	7938	1000	1000

**Table 3:** Experimental conditions for lateralization.

Condition	Strategy	Amplitude Modulation on High-Rate Channels	ITD Provided
1	All-high CIS	125 Hz	Envelope
2	Mixed-rate	125 Hz	Envelope + Pulse
3	Mixed-rate	0 Hz	Pulse

**Table 4:** Experimental conditions for speech testing.

Strategy	MAP	ITDs	Condition
ACE	Clinical	0 μs	Quiet
CIS	Ten-channel	0, +800 μs	Quiet, −3, 0, +3 dB
Mixed-rate	Ten-channel	0, +800 μs	Quiet, −3, 0, +3 dB

## Data Availability

Data and contents are available upon request by contacting the final author of the manuscript.
